# Effects of supervised high-intensity interval training on motivational outcomes in men with prostate cancer undergoing active surveillance: results from a randomized controlled trial

**DOI:** 10.1186/s12966-022-01365-2

**Published:** 2022-09-29

**Authors:** Dong-Woo Kang, Normand G. Boulé, Catherine J. Field, Adrian S. Fairey, Kerry S. Courneya

**Affiliations:** 1 Department of Medical Oncology, Dana-Farber Cancer Institute, Boston, MA USA; 2 Department of Medicine, Harvard Medical School, Boston, MA USA; 3 Faculty of Kinesiology, Sport, and Recreation, University of Alberta, Edmonton, AB Canada; 4 Department of Agricultural, Food, and Nutritional Science, Faculty of Agricultural, Life and Environmental Sciences, University of Alberta, Edmonton, AB Canada; 5 Division of Urology, Department of Surgery, Faculty of Medicine & Dentistry, University of Alberta, Edmonton, AB Canada

**Keywords:** Exercise motivation, High-intensity interval training, Supervised exercise, Prostate cancer, Active surveillance, Randomized controlled trial

## Abstract

**Background:**

Understanding the motivational effects of supervised aerobic high-intensity interval training (HIIT) may help men with prostate cancer undergoing active surveillance initiate and maintain exercise behavior, however, few studies have addressed this question. This report explored exercise motivation in men with prostate cancer undergoing active surveillance participating in a randomized exercise trial.

**Methods:**

The Exercise during Active Surveillance for Prostate Cancer (ERASE) trial randomized 52 men with prostate cancer on active surveillance to the HIIT exercise group or the usual care (UC) group. The exercise program was supervised aerobic HIIT conducted three times per week for 12 weeks. The motivation questions were developed using the Theory of Planned Behavior and included motivational constructs, anticipated and experienced outcomes, and barriers to HIIT during active surveillance.

**Results:**

The HIIT group attended 96% of the planned exercise sessions with 100% compliance to the exercise protocol. Motivation outcome data were obtained in 25/26 (96%) participants in the HIIT group and 25/26 (96%) participants in the UC group. At baseline, study participants were generally motivated to perform HIIT. After the intervention, the HIIT group reported that HIIT was even more enjoyable (*p* < 0.001; *d* = 1.38), more motivating (*p* = 0.001; *d* = 0.89), more controllable (*p* < 0.001; *d* = 0.85), and instilled more confidence (*p* = 0.004; *d* = 0.66) than they had anticipated. Moreover, compared to UC, HIIT participants reported significantly higher perceived control (*p* = 0.006; *d* = 0.68) and a more specific plan (*p* = 0.032; *d* = 0.67) for performing HIIT over the next 6 months. No significant differences were found in anticipated versus experienced outcomes. Exercise barriers were minimal, however, the most often reported barriers included pain or soreness (56%), traveling to the fitness center (40%), and being too busy and having limited time (36%).

**Conclusion:**

Men with prostate cancer on active surveillance were largely motivated and expected significant benefits from a supervised HIIT program. Moreover, the men assigned to the HIIT program experienced few barriers and achieved high adherence, which further improved their motivation. Future research is needed to understand long-term exercise motivation and behavior change in this setting.

**Trial registration:**

Clinicaltrials.gov, NCT03203460. Registered on June 29, 2017.

**Supplementary Information:**

The online version contains supplementary material available at 10.1186/s12966-022-01365-2.

## Introduction

Low-to-moderate grade prostate cancers are often indolent and managed by active surveillance, where patients can avoid immediate invasive treatment and are regularly monitored for any signs of disease progression. Active surveillance enables these patients to avoid potentially unnecessary immediate treatment side effects, however, it does require regular monitoring including prostate-specific antigen (PSA) testing (e.g., every 6 months) and prostate biopsy, digital rectal exam, and/or multi-parametric magnetic resonance imaging (e.g., every 12-18 months or as indicated). Unfortunately, one in three men on active surveillance eventually experience disease progression and undergo invasive treatment such as surgery or radiation therapy, and are at risk for other chronic comorbidities such as cardiovascular disease and metabolic disorders [1]. We recently completed a phase II randomized controlled trial called the Exercise during Active Surveillance for Prostate Cancer (ERASE) trial [2]. In the primary paper, we reported that a 12-week, thrice weekly, aerobic high-intensity interval training (HIIT) program significantly increased cardiorespiratory fitness and suppressed biomarkers related to prostate cancer progression [3] compared to usual care. In a secondary paper, we reported HIIT reduced prostate cancer-specific anxiety and fear of cancer progression, perceived stress, and fatigue, and improved self-esteem [4].

Our findings suggest that supervised HIIT would be advantageous for a broader community of prostate cancer patients on active surveillance to gain various physical, psychological, and clinical benefits. Understanding the motivational effects of supervised HIIT would provide important information for exercise specialists to help these patients initiate and maintain exercise behavior. Previous research has demonstrated the motivational value of supervised exercise in various cancer patient groups [5–10]; however, no study to date has focused on prostate cancer patients on active surveillance who are generally older men with a very favorable cancer prognosis receiving no cancer treatments but with substantial comorbidities (i.e., > 60% having one or more health conditions other than cancer). These distinct patient characteristics may influence the motivational response to supervised exercise. Moreover, research has also suggested that the motivational value of supervised exercise may vary depending on the exercise program [5, 7, 11]. To our knowledge, no study to date has examined the motivational effects of HIIT in any cancer patient group.

The purpose of this paper was to report the secondary outcomes of the ERASE trial related to the motivational impact of the supervised HIIT program. Similar to previous research [5, 7–11], we adopted the Theory of Planned Behavior (TPB) [12, 13] to assess motivational outcomes, which is a social cognitive framework that focuses on intention (motivation) as the primary determinant of human behavior. In this model, motivation is an indicator of how strong the person’s intention is to perform the behavior, and it is affected by four components: (1) instrumental attitude (i.e., anticipated benefits from the behavior), (2) affective attitude (i.e., anticipated enjoyment of the behavior), (3) perceived behavioral control (i.e., perceived difficulty, controllability, and confidence in performing the behavior), and (4) subjective norm (i.e., anticipated external support for the behavior). Based on previous research [5, 7–11], we hypothesized that men with prostate cancer undergoing active surveillance assigned to the supervised HIIT program would experience positive motivational changes for exercise compared to baseline and to the usual care (UC) group.

## Methods

### Study design and population

Detailed study methods of the ERASE Trial have been published elsewhere [2, 3]. The ERASE Trial was a randomized controlled trial examining the effects of a 12-week aerobic HIIT program in prostate cancer patients on active surveillance conducted at the University of Alberta, Edmonton, Canada. The trial was registered in clinicaltrial.gov (NCT03203460) and approved by the Health Research Ethics Board of Alberta – Cancer Committee (HREBA.CC-17-0248). All participants provided written consent for study participation and blood banking. Participants were recruited from the Kaye Edmonton Clinic. Eligibility criteria included 18 years of age or older, diagnosed with prostate cancer and undergoing active surveillance, no plan for curative treatment at the time of recruitment, no contraindications for performing cardiopulmonary fitness testing and high-intensity aerobic training, and no participation in any structured vigorous-intensity exercise. Eligible patients were briefly informed about the study by their urologists during their monitoring visits and, if interested, were referred to the study coordinator for details about the study, further screening, and study enrollment. Once patients agreed to participate in the study, baseline blood draw and fitness assessments were completed and participants were randomized either to HIIT or UC.

### Exercise intervention

Details of the study interventions have been described elsewhere [2]. In brief, the HIIT group was given a supervised, thrice-weekly, aerobic HIIT program for 12 weeks conducted at our exercise facility within 5 minutes walking distance from the cancer center. Each HIIT session comprised 2 minutes of high-intensity exercise (workload corresponding to 85-95% peak oxygen consumption [VO_2peak_]) followed by 2 minutes of light-intensity exercise recovery (workload corresponding to 40% VO_2peak_), with progression from 5 to 8 intervals resulting in 28 minutes to 40 minutes of exercise (including warm-up and cool-down for 5 minutes each). Participants in the UC group were asked not to begin any structured high-intensity exercise during the intervention period (12 weeks). They were then offered a 4-week HIIT program at our facility or a 12-week community-based exercise program.

### Outcome assessment

#### Motivational outcomes

Anticipated motivation for the HIIT exercise program was measured at baseline for both groups. Experienced motivation of the HIIT exercise program was measured at postintervention only for the HIIT group. Exercise motivation outcomes were based on the TPB and included the constructs of intention, self-efficacy, perceived behavioral control, instrumental and affective attitudes, and subjective norms. We used the TPB items recommended by Ajzen [12]. Each TPB construct was assessed by a corresponding question using a 5-point Likert scale (i.e., 1 = not at all, 2 = a little bit, 3 = somewhat, 4 = quite a bit, and 5 = very much). Specifically, we asked how (1) ‘beneficial’ the HIIT exercise program will be/was (instrumental attitude), (2) ‘enjoyable’ the HIIT exercise program will be/was (affective attitude), (3) ‘supportive’ their family/friends will be/were of them doing the HIIT exercise program (subjective norm), (4) ‘motivated’ they are/were to do the HIIT exercise program (intention), (5) ‘confident’ they are/were to do the HIIT exercise program (self-efficacy), (6) and ‘controllable’ and ‘difficult’ the program will be/was for them (perceived behavioral control). At baseline (prior to randomization), all patients were asked their ‘anticipated’ exercise motivation if they were assigned to the 12-week HIIT intervention during active surveillance (e.g., how beneficial will the program be). At post-intervention, patients in the HIIT group were asked to reflect on their ‘experienced’ motivation during the past 12 weeks of the intervention period (e.g., how beneficial was the program). Also at postintervention, both the HIIT and UC groups were asked about their exercise motivation for the next 6 months after the study completion which included an additional item about their exercise plan (i.e., “Do you have a specific plan for where, when, and how you are going to do exercise over the next 6 months?”).

#### Anticipated and experienced outcomes

Anticipated and experienced outcomes (i.e., behavioral beliefs) of the HIIT exercise program were measured using a questionnaire at baseline for both groups and at postintervention for the HIIT group only. The questionnaire consisted of 12 items asking about outcomes identified from the previous literature [9] with additional questions relevant to prostate cancer patients on active surveillance (e.g., growth of prostate cancer and PSA levels). A 7-point Likert scale was used ranging from − 3 (very much worse) through 0 (no chance) to 3 (very much better) for all items except for asking about the chance that they will need prostate cancer treatments and PSA levels (from − 3 being higher to 3 being lower). At baseline, all participants were asked what effects they thought the HIIT program would have on outcomes (i.e., anticipated outcomes). At postintervention, only the intervention group was asked what effects the HIIT program actually had on those outcomes (i.e., experienced outcomes). At postintervention, we did not ask about outcomes that could not be directly observed by the participants (e.g., immune function, PSA levels).

#### Experienced barriers

Perceived barriers (i.e., control beliefs) to the HIIT program were assessed using a 14-item questionnaire at postintervention for the HIIT group. The questionnaire was comprised of questions asking about barriers to exercise they experienced over the past 12-week intervention. The barriers in the questionnaire were based on those reported in previous exercise oncology trials [9, 14–16] with additional potential barriers patients might have experienced (e.g., prostate-related symptoms, fear/worry of cancer progressing/spreading). A 7-point Likert scale was used ranging from 1 (not at all) to 7 (very much).

### Covariates

Demographic and behavioral characteristics were collected at baseline using a set of self-reported questions including age, race, marital status, employment status, education level, income, alcohol consumption, and smoking. Current exercise behavior (i.e., exercise levels for the past month at the time of recruitment) was assessed using the Godin Leisure Time Exercise Questionnaire [17]. Medical outcomes, including pathological and clinical profiles of prostate cancer (i.e., tumor stage, Gleason grade, PSA levels, and time on active surveillance) and comorbidities, were obtained via electronic medical records.

### Statistical analysis

Changes in anticipated versus experienced motivational outcomes before and after the intervention within the HIIT group were analyzed using the paired t-test. Between-group mean differences in motivation outcomes for performing HIIT over the next 6 months measured at postintervention were analyzed using univariate analyses adjusting for baseline values of the outcome and two variables that were not balanced between groups at baseline (i.e., marital status and employment status). Specific Plan was not measured at baseline and adjusted only for marital status and employment status. The effect size was calculated for changes in motivational outcomes using Cohen’s d [18] by dividing the mean change/difference with the pooled standard deviation at baseline, where d = 0.2, 0.5, and 0.8 were considered small, medium, and large effects, respectively. Experienced barriers to exercise were reported as categories by the percentages of patients who experienced each barrier (i.e., Not at all = score 1, Somewhat = score 2-4, and Quite/Very much = score 5-7). SPSS version 26 (SPSS Inc., Chicago, IL, USA) was used for statistical analyses.

## Results

Participant flow in the ERASE trial has been reported elsewhere [3]. In brief, a total of 52 men with prostate cancer undergoing active surveillance were randomized to the HIIT (*n* = 26) or UC (*n* = 26) group. Motivation outcome data were obtained in 25/26 (96%) participants in the HIIT group and 25/26 (96%) participants in the UC group. The HIIT group attended 96% of the planned exercise sessions with 100% compliance to the exercise protocol.

Baseline demographic, behavioral, and medical profiles of the ERASE participants have been described elsewhere [3]. In brief, the mean age was 63.4 ± 7.1, 89% Caucasian, 63% employed, 71% married, 39% completed university/college, 2% were current smokers, 12% regular drinkers, and the average time spent in moderate-intensity exercise was 61 ± 99 minutes per week. The mean body mass index was 29.0 ± 4.7, 83% had comorbidities (e.g., 60% arthritis/arthralgia and 31% hypertension), 90% were T1c stage prostate cancer, 96% Gleason grade of 6, the mean PSA level was 7.3 ± 3.2, and the mean time since starting active surveillance was 1.9 ± 2.2 years. Marital status (married/common-law vs. divorced/separated/never married/widowed) and employment status (full-time/part-time vs. retired/homemaker/disability/sick leave) were not balanced between groups (HIIT: 65% vs. UC: 77% [*p* = 0.034] and HIIT: 48% vs. UC: 77% [*p* = 0.026], respectively) and were adjusted for in the analyses.

Table 1 describes the anticipated motivation and anticipated outcomes of supervised HIIT at baseline for all participants. Overall, participants expected that the HIIT program would be quite beneficial (4.2 ± 0.7), somewhat/quite enjoyable (3.7 ± 0.9), somewhat difficult (2.8 ± 0.9), somewhat/quite controllable (3.7 ± 0.8), and they were quite confident (4.2 ± 0.8), quite motivated (4.2 ± 0.8), and felt they would be quite/very much supported by family/friends (4.5 ± 0.6). In terms of anticipated outcomes, all outcomes were expected to improve after HIIT (i.e., > 0 on a − 3 to + 3 scale) but especially physical fitness (2.4 ± 0.8), quality of life (2.1 ± 0.9), the immune system’s ability to fight cancer (1.9 ± 0.9), length of survival (1.8 ± 1.1), and preparation for prostate cancer treatment (1.6 ± 1.2).

**Table 1:** Baseline motivation and anticipated outcomes of high-intensity interval training in men with prostate cancer undergoing active surveillance in the ERASE trial

Variables	Overall (***N*** = 52)	HIIT (***N*** = 26)	UC (***N*** = 26)
*Motivational outcomes*^b^			
Beneficial	4.2 (0.7)	4.2 (0.7)	4.2 (0.8)
Enjoyable	3.7 (0.9)	3.4 (0.7)	3.9 (0.9)
Difficult	2.8 (0.9)	2.8 (0.9)	2.7 (1.0)
Controllable	3.7 (0.8)	3.5 (0.8)	3.9 (0.8)
Confident	4.2 (0.8)	4.0 (0.7)	4.3 (0.8)
Motivated	4.2 (0.8)	4.0 (0.8)	4.4 (0.8)
Supported	4.5 (0.6)	4.4 (0.6)	4.6 (0.6)
*Anticipated outcomes*^c^			
Physical fitness	2.4 (0.8)	2.4 (0.6)	2.5 (0.9)
Quality of life	2.1 (0.9)	2.0 (0.9)	2.2 (0.9)
Immune system’s ability to fight cancer	1.9 (0.9)	2.0 (0.9)	1.9 (1.0)
Length of survival	1.8 (1.1)	1.8 (0.9)	1.7 (1.2)
Preparation for prostate cancer treatment	1.6 (1.2)	1.7 (1.2)	1.6 (1.2)
Sense of control over prostate cancer	1.2 (1.1)	1.0 (1.1)	1.4 (1.0)
Chance that prostate cancer treatments are needed^a^	1.1 (1.1)	1.0 (1.1)	1.2 (1.1)
Growth of prostate cancer	1.1 (1.2)	0.9 (1.2)	1.2 (1.2)
PSA levels^a^	1.1 (1.1)	0.9 (1.0)	1.3 (1.2)
Stop thinking about prostate cancer	1.0 (1.1)	0.7 (1.1)	1.3 (1.1)
Fear/worry of prostate cancer progressing	1.0 (1.1)	0.9 (1.1)	1.1 (1.1)
Aggressiveness of prostate cancer	1.0 (1.2)	0.8 (1.1)	1.1 (1.3)

Values are mean (SD). HIIT, high-intensity interval training; UC, usual care; PSA, prostate-specific antigen

^a^ Higher values indicate lower chance or lower PSA levels

^b^Motivational outcomes were assessed on a 5-point scale from 1 (not at all) to 5 (very much)

^c^Anticipated outcomes were assessed on a 7-point scale from −3 (very much worse) to 0 (no change) to + 3 (very much better)

### Motivation outcomes

**Fig. 1:**
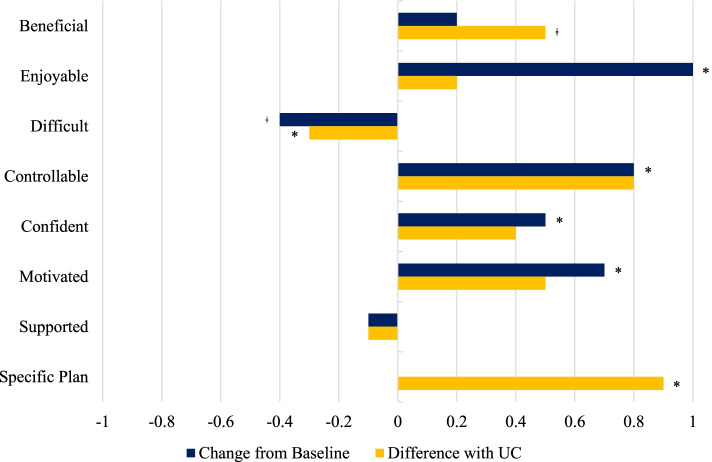
Motivational outcomes in the HIIT group at postintervention in the ERASE trial. Note: “Change from baseline” refers to motivation for the previously completed 12-week supervised HIIT program; “Difference with UC” refers to motivation for the HIIT program over the next 6 months. HIIT = high intensity interval training; UC = usual care. *p < 0.05; ǂ*p* < 0.10

Table 2 and Fig. 1 show the effects of the supervised HIIT program on exercise motivation among the participants in the HIIT group. Participants experienced, compared to what they had anticipated, that the supervised HIIT program was more enjoyable (mean change, 1.0; 95% confidence interval [CI], 0.6 to 1.4; *p* < 0.001; *d* = 1.38), more motivating (mean change, 0.7; 95% CI, 0.3 to 1.0; *p* = 0.001; *d* = 0.89), more controllable (mean change, 0.8; 95% CI, 0.4 to 1.1; *p* < 0.001; *d* = 0.85), and instilled more confidence (mean change, 0.5; 95% CI, 0.2 to 0.9; *p* = 0.004; *d* = 0.66). Table 3 and Fig. 1 show the effects of the supervised HIIT program on motivation for performing HIIT over the next 6 months assessed at post-intervention in both groups. Compared to UC, HIIT participants reported significantly higher controllability (adjusted between-group mean difference, 0.8; 95% CI, 0.2 to 1.3; *p* = 0.006; *d* = 0.68), a more specific plan (adjusted between-group mean difference, 0.9; 95% CI, 0.1 to 1.8; *p* = 0.032; *d* = 0.67), and a borderline significantly higher anticipated benefit (adjusted between-group mean difference, 0.5; 95% CI, 0.0 to 1.0; *p* = 0.065; *d* = 0.55) of performing HIIT over the next 6 months.

**Table 2:** Effects of high-intensity interval training on exercise motivation in 25 men with prostate cancer undergoing active surveillance randomized to the exercise intervention in the ERASE trial

Variables	Baseline (Anticipated)		Postintervention (Experienced)		Mean change			
	Mean	SD	Mean	SD	Mean	95% CI	*p*	*d*
Beneficial	4.2	0.7	4.3	0.9	0.2	−0.2 to 0.6	0.31	0.18
Enjoyable	3.4	0.7	4.4	0.8	1.0	0.6 to 1.4	< 0.001	1.38
Difficult	2.8	0.9	2.4	0.9	− 0.4	−0.9 to 0.0	0.053	−0.50
Controllable	3.5	0.8	4.2	0.9	0.8	0.4 to 1.1	< 0.001	0.85
Confident	4.0	0.7	4.5	0.7	0.5	0.2 to 0.9	0.004	0.66
Motivated	4.0	0.8	4.6	0.6	0.7	0.3 to 1.0	0.001	0.89
Supported	4.4	0.6	4.3	0.9	−0.1	− 0.4 to 0.2	0.58	−0.10

Motivational outcomes were assessed on a 5-point scale from 1 (not at all) to 5 (very much)

**Table 3:** Effects of high-intensity interval training on motivation for performing high intensity interval training over the next 6 months in men with prostate cancer undergoing active surveillance after the ERASE trial

Variables	Mean	SD	Adjusted between-group difference^a^			
			Mean	95% CI	*p*	*d*
Beneficial						
HIIT (*n* = 25)	4.3	0.8	0.5	0.0 to 1.0	0.065	0.55
UC (*n* = 25)	3.8	0.9				
Enjoyable						
HIIT (*n* = 25)	3.8	1.2	0.2	−0.4 to 0.9	0.43	0.08
UC (*n* = 25)	3.7	0.8				
Difficult						
HIIT (*n* = 25)	2.2	1.0	−0.3	−0.8 to 0.3	0.32	−0.40
UC (*n* = 25)	2.6	1.0				
Controllable						
HIIT (*n* = 25)	4.2	0.9	0.8	0.2 to 1.3	0.006	0.68
UC (*n* = 25)	3.6	0.9				
Confident						
HIIT (*n* = 25)	4.0	1.0	0.4	−0.2 to 1.0	0.21	0.21
UC (*n* = 25)	3.8	1.0				
Motivated						
HIIT (*n* = 25)	4.1	1.0	0.5	−0.1 to 1.1	0.11	0.30
UC (*n* = 25)	3.8	0.9				
Supported						
HIIT (*n* = 25)	4.1	1.2	−0.1	−0.6 to 0.7	0.87	−0.12
UC (*n* = 25)	4.3	0.8				
Specific Plan						
HIIT (*n* = 25)x	3.7	1.3	0.9	0.1 to 1.8	0.032	0.67
UC (*n* = 25)	2.8	1.5				

Motivational outcomes were assessed on a 5-point scale from 1 (not at all) to 5 (very much)

^a^Between-group mean difference was adjusted for marital status, employment status, and baseline value of the outcome, except for Specific Plan which was adjusted only for marital status and employment status as this outcome was not measured at baseline

### Anticipated/experienced outcomes

Table 4 shows the differences in anticipated outcomes at baseline versus experienced outcomes at postintervention among participants in the HIIT group. No significant differences were found in what was anticipated versus experienced for physical fitness, quality of life preparation for prostate cancer treatment, sense of control over prostate cancer, chance that prostate cancer treatments are needed, fear/worry of prostate cancer progressing, and thinking about prostate cancer.

**Table 4:** Differences in anticipated versus experienced outcomes of high-intensity interval training in 25 men with prostate cancer undergoing active surveillance randomized to the exercise intervention in the ERASE trial

Variables	Baseline (Anticipated)		Postintervention (Experienced)		Mean change			
	Mean	SD	Mean	SD	Mean	95% CI	*p*	*d*
Physical fitness	2.4	0.6	2.4	0.8	0.0	−0.4 to 0.4	1.0	−0.04
Quality of life	2.0	0.9	1.9	1.1	−0.1	−0.7 to 0.4	0.63	−0.12
Preparation for prostate cancer treatment	1.7	1.2	1.5	1.3	−0.1	− 0.6 to 0.3	0.59	− 0.14
Sense of control over prostate cancer	1.0	1.1	1.1	1.3	0.0	−0.4 to 0.5	0.87	0.07
Chance that prostate cancer treatments are needed^a^	1.0	1.1	1.0	1.2	−0.1	−0.6 to 0.4	0.73	−0.03
Fear/worry of prostate cancer progressing	0.9	1.1	0.8	1.2	−0.2	−0.5 to 0.2	0.36	−0.08
Stop thinking about prostate cancer	0.7	1.1	1.1	1.3	0.4	−0.1 to 0.9	0.12	0.36

^a^ Higher values indicate lower chance. Anticipated outcomes were assessed on a 7-point scale from −3 (very much worse) to 0 (no change) to + 3 (very much better)

### Perceived barriers to HIIT

Table 5 shows perceived barriers to supervised HIIT among participants in the HIIT group assessed at postintervention. Overall, the mean scores of each exercise barrier ranged from 1.0 to 1.9 using a 1 (not at all) to 7 (very much) point Likert scale. The exercise barriers that participants most often reported to be at least somewhat (≥2) of a barrier included pain or soreness (56%), traveling to the fitness center (40%), being too busy and having limited time (36%), feeling tired or fatigued (28%), and the exercise program being too demanding/difficult (28%).

**Table 5:** Perceived barriers to high-intensity interval training in 25 men with prostate cancer undergoing active surveillance randomized to the exercise intervention in the ERASE trial

Variables	Mean	SD	Percentage distribution		
			Not at all (1)	Somewhat (2-4)	Very much (5-7)
Pain or soreness	1.9	1.1	44.0%	52.0%	4.0%
Too busy and had limited time	1.8	1.3	64.0%	28.0%	8.0%
Travelling to the fitness centre	1.8	1.2	60.0%	32.0%	8.0%
Feeling tired or fatigued	1.4	0.7	72.0%	28.0%	0.0%
Exercise program too demanding/difficult	1.4	0.7	72.0%	28.0%	0.0%
Bad weather	1.3	0.9	84.0%	12.0%	4.0%
Feeling sick or not feeling well	1.3	0.6	76.0%	24.0%	0.0%
Fear/worry of cancer progressing/spreading	1.2	0.5	87.5%	12.5%	0.0%
Urinary incontinence	1.2	0.6	84.0%	16.0%	0.0%
Sexual problems	1.2	1.2	96.0%	0.0%	4.0%
Lack of motivation	1.2	0.4	76.0%	24.0%	0.0%
Bowel problems	1.1	0.3	88.0%	12.0%	0.0%
Medical appointments	1.1	0.3	88.0%	12.0%	0.0%
Having prostate cancer	1.0	0.2	96.0%	4.0%	0.0%

Experienced barriers were assessed on a 7-point scale from 1 (not at all) to 7 (very much)

## Discussion

Overall, men with prostate cancer undergoing active surveillance in the ERASE trial were quite motivated, confident, and felt supported for performing a supervised aerobic HIIT program. They anticipated that HIIT would be somewhat/quite beneficial, enjoyable, difficult, and controllable. After completing the 12-week HIIT program with a high level of adherence, the participants in the HIIT group experienced HIIT as more enjoyable, more motivating, more controllable, and provided more confidence than anticipated. Furthermore, the HIIT group reported higher perceived control and a more specific plan for doing HIIT after the completion of the study compared to the UC group. Finally, exercise barriers were minimal during the intervention period, however, the HIIT group reported pain or soreness, traveling to the exercise facility, and having limited time to be the most common constraints to attending HIIT sessions.

To our knowledge, our study is the first to report motivational outcomes for HIIT in cancer patients. Previous studies have documented that patients diagnosed with various types of cancer, including breast [5, 19, 20], colorectal [6, 21], prostate [7, 20], endometrial [22], bladder [23], brain [24], lung [8], and rectal cancer [9], were generally motivated to participate in moderate-to-vigorous intensity exercises. Participants in the ERASE trial were somewhat/quite motivated about participating in HIIT at baseline. Based on the TPB constructs, anticipated external support for doing HIIT (i.e., subjective norm) was the strongest motivational construct (4.5 points out of 5.0), followed by anticipated benefits of HIIT (i.e., instrumental attitude), being motivated about doing HIIT (i.e., intention), and being confident about doing HIIT (self-efficacy). Given the increase in HIIT interventions in exercise oncology trials [25], our findings for the first time suggest that cancer patients are also motivated to participate in higher-intensity exercise which is generally assumed to be more challenging or demanding. However, it is unknown if cancer patients would prefer HIIT over lower-intensity exercises. For example, Courneya et al., compared different exercise modalities and reported that breast cancer patients receiving chemotherapy preferred and were more motivated for combined aerobic and resistance exercise compared to high-dose or low-dose aerobic exercise only [11]. Future studies focusing on comparisons in motivation for difference exercise intensities (HIIT vs. moderate-intensity continuous training [MICT]) will be beneficial for prescribing and disseminating exercise programs in cancer patients.

In addition, patients anticipated improvements in all listed outcomes at baseline, with physical (i.e. physical fitness) and psychological health (i.e., quality of life) being expected to improve the most. Interestingly, patients on active surveillance also anticipated improvements in cancer-related or even clinical outcomes, such as immune system’s ability to fight cancer, length of survival, and preparation for prostate cancer treatment. These outcomes may be critical considering patients’ unique concerns and anxiety about their existing untreated tumor and its potential progression [26–28]. Nevertheless, it should be noted that our study participants had interest in exercise in general at recruitment as they were self-motivated to participate in the exercise trial. Moreover, it is important to note that study participants were not on any treatments for their cancer at the time of the HIIT intervention and were also otherwise quite healthy.

After the HIIT intervention, participants assigned to HIIT found the program significantly more enjoyable than they anticipated (effect size *d* = 1.38). This finding is in line with previous literature reporting exercise was more enjoyable than expected including in breast [11], lymphoma [10], lung [8], and rectal cancer patients [9], while patients in a few other studies, including endometrial [29] and bladder cancer [23], reported that exercise was less enjoyable. It is worth considering the discrepancies in enjoyableness of exercise given various factors such as cancer type, treatment trajectories, and exercise prescriptions. Our finding is the first evidence that a HIIT program could be more enjoyable than anticipated among cancer patients. Along with other studies showing enjoyment of HIIT compared to MICT in various populations [30–32], our finding supports the use of HIIT as an exercise modality among cancer patients that could exert not only superior health benefits [33] but also greater enjoyableness and potentially higher adherence [32, 34].

Furthermore, along with enjoyableness, other motivational outcomes, including controllability, confidence, and motivation about HIIT were higher compared to their anticipation at baseline. These findings overall are consistent with previous studies reporting that cancer patients experienced more positive motivational outcomes than expected [5, 7–11]. On the other hand, there were no significant differences in anticipated versus experienced benefits of HIIT or support for participating in HIIT from other people. These non-significant changes might be due to the high level of anticipated benefits and support at baseline, which thus could have led to ceiling effects on these outcomes.

Regarding the participants’ perception of doing HIIT on their own after the study period, the HIIT group reported higher anticipated controllability and a more specific plan compared to the UC group. These findings suggest that participating in the 12-week supervised HIIT program was sufficient to provide patients with a better understanding and details about the HIIT program compared to their abstract conception of HIIT that they might have had at baseline. It should also be noted that the professionally supervised exercise intervention setting in our study likely helped patients lower or eliminate any potential concerns of possible injury with participating in HIIT [35, 36] particularly given that most patients had one or more baseline health conditions (i.e., > 60%) [3]. Furthermore, having controllability and a specific plan may portend the sustainability and scalability of the intervention where self-directed exercise (i.e., non-supervised, home-based or community-based) may be necessary. Nevertheless, intention to continue HIIT on their own in the exercise group does not guarantee the continuation of exercise given the well-known intention-behavior gap for exercise in both healthy [37] and cancer [38] populations. Further follow-up on exercise behavior after the study period is necessary to know whether their improved motivational outcomes such as controllability and planning would translate into sustained exercise participation.

Interestingly, there were no significant differences between anticipated versus experienced outcomes from HIIT, however, patients anticipated substantial benefits for many outcomes. These data suggest that HIIT largely delivered the positive benefits that these men were expecting. In fact, we found that the objective measure of physical fitness (i.e., VO_2peak_) significantly improved in the HIIT group compared to the UC group [3]. Similarly, quality of life was assessed using a validated questionnaire (i.e., the European Organization for Research and Treatment of Cancer – Quality of Life-C30), and borderline significantly improved in the HIIT group compared to the UC group [4]. It is interesting that some patients reported improved prostate cancer and/or active surveillance-related outcomes were both anticipated and experienced, such as better preparation for prostate cancer treatment, sense of control over prostate cancer, or chance that prostate cancer treatment is needed. Again, such positive perceived outcomes can indicate that exercise, particularly HIIT, may relieve psychological distress or concerns that active surveillance patients experience, which may help a subset of patients with significant anxiety or fear of cancer progression remain on active surveillance [39]. However, although the ERASE trial found a significant reduction in biochemical progression of prostate cancer [3] and other epidemiological studies suggested the association between physical activity and clinical outcomes (e.g., cancer reclassification or active surveillance discontinuation) [40, 41], it is still unknown if exercise can yield long-term clinical benefits.

Patients in the HIIT group reported minimal barriers to HIIT (ranging from 1.0 to 1.9 using a 7-point scale), which is unsurprising given the very high intervention adherence (96%) and compliance (100%) in the ERASE trial [3]. Nevertheless, the most commonly reported barrier to HIIT was pain or soreness. The sources of pain or soreness were not specifically reported, however, joint pain or arthritis may likely be the primary reason, considering 62% of patients in the HIIT group already had arthritis or arthralgia at baseline, and six cases of joint pain were reported as adverse events during the HIIT intervention [3]. This finding is in contrast to other studies reporting ‘too busy’ in general [42] or ‘treatment side effects’ during treatment [43] as the most common barriers to exercise among prostate cancer patients. Future studies can consider other exercise modalities other than a treadmill (e.g., bike) that can prevent potential exacerbation of pre-existing joint or age-related musculoskeletal issues given the older age of the prostate cancer population.

Active surveillance patients are situated in a unique clinical setting where they are offered neither ‘treatment’ for the tumor nor any guidance to help suppress or delay cancer growth, reduce anxiety/fear, or prepare for possible treatment. The importance of participating in exercise during active surveillance has been highlighted in recent studies where exercise can exert physical and psychological health benefits as well as potential clinical benefits [3, 40, 41, 44, 45]. The ERASE trial suggests that a particular exercise program (i.e., HIIT) not only improved physical fitness, biochemical outcomes, and patient-reported outcomes but also improved motivational outcomes in prostate cancer patients on active surveillance. Further studies are needed to identify whether motivational outcomes will impact long-term exercise behavior in these populations and which behavior strategies would maximize participation in HIIT beyond the supervised intervention. Moreover, future studies that focus on identifying whether these motivational outcomes mediate the effects of supervised exercise on long term exercise behavior change would be beneficial (e.g., mediation analyses or structural equation modeling).

The key strengths of the current study include (1) the first study to report exercise motivation and barriers in men with prostate cancer on active surveillance, (2) the comprehensive assessments of motivational outcomes using a validated framework of human behavior (i.e., TPB), (3) the assessment of anticipated and experienced outcomes, and (4) the randomized controlled design with usual care that allows comparisons of motivational outcomes between the exercise and control groups. Limitations include (1) a lack of longer-term follow-up of exercise behavior, (2) a likely highly motivated sample (i.e., self-selected) lacking generalizability to those who are not interested or motivated to exercise or who were not able to participate due to chronic or medical conditions, (3) a well-educated, higher income, and racially homogeneous sample that limits the generalizability of our findings to less advantaged groups, and (4) limited power to detect potentially meaningful effects (e.g., d = 0.33 to 0.50) on these secondary motivational outcomes.

## Conclusion

As expected, ERASE trial participants were highly motivated and had positive expectations about participating in HIIT during active surveillance. Moreover, participants randomized to supervised HIIT experienced few exercise barriers and achieved a high level of adherence, which further improved their motivation for HIIT. Our findings may have important implications for practice. First, our findings may be used to increase recruitment to supervised HIIT programs in prostate cancer patients on active surveillance by informing them that HIIT is likely to be more enjoyable, controllable, and motivating than they anticipate. Second, our findings suggest that 12 weeks of supervised HIIT during active surveillance for prostate cancer may further improve exercise motivation, and potentially lead to longer-term exercise adherence [46]. Future large phase trials should address the generalizability of our findings by recruiting a more diverse and vulnerable population (e.g., less educated, lower income, racially and ethnically diverse, more sedentary, poorer health-related fitness) and incorporate long-term follow-up of motivational outcomes, exercise behavior, and clinical outcomes that will ultimately translate to broader clinical and community practice.

## Supplementary Information

**Additional file 1.** Supplementary material-CONSORT diagram.

## Data Availability

The datasets generated and/or analysed during the current study are not publicly available but are available from the corresponding author on reasonable request.

## Abbreviations

CI: Confidence interval
ERASE: Exercise during Active Surveillance for Prostate Cancer
HIIT: High-intensity interval training
MICT: Moderate-intensity continuous training
PSA: Prostate-specific antigen
TPB: Theory of Planned Behavior
UC: Usual care
VO_2peak_: Peak oxygen consumption
