# Supplementary figures and images for: Effects of supervised high-intensity interval training on motivational outcomes in men with prostate cancer undergoing active surveillance: results from a randomized controlled trial
Source: Int J Behav Nutr Phys Act. 2022 Sep 29;19:126. doi: 10.1186/s12966-022-01365-2 (PMC9524100; doi:10.1186/s12966-022-01365-2)

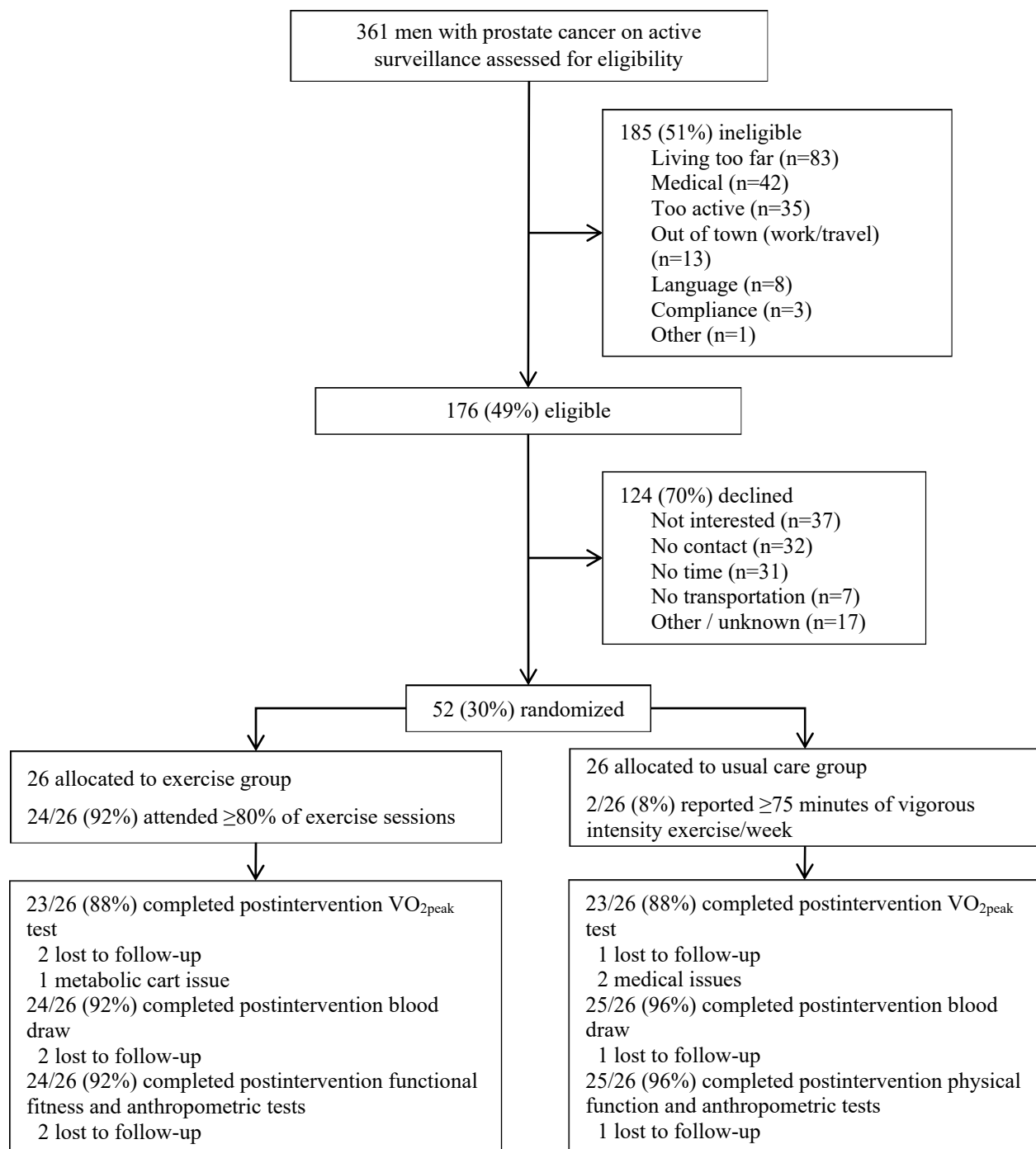

Supplement: Supplementary file 1 — Additional file 1. Supplementary material-CONSORT diagram. [file 12966_2022_1365_MOESM1_ESM.pdf]
